# Restricted spirometry and cardiometabolic comorbidities: results from the international population based BOLD study

**DOI:** 10.1186/s12931-022-01939-5

**Published:** 2022-02-17

**Authors:** Katarzyna Kulbacka-Ortiz, Filip J. J. Triest, Frits M. E. Franssen, Emiel F. M. Wouters, Michael Studnicka, William M. Vollmer, Bernd Lamprecht, Peter G. J. Burney, Andre F. S. Amaral, Lowie E. G. W. Vanfleteren

**Affiliations:** 1 COPD Center, Department of Respiratory Medicine and Allergology, Sahlgrenska University Hospital, Gothenburg, Sweden; 2 Department of Internal Medicine and Clinical Nutrition, Institute of Medicine, Sahlgrenska Academy, University of Gothenburg, Gothenburg, Sweden; 3 CIRO, Centre of Expertise for Chronic Organ Failure, Horn, the Netherlands; 4 Department of Respiratory Medicine, AZ Sint-Lucas, Gent, Belgium; 5 Department of Respiratory Medicine, MUMC+, Maastricht University Medical Centre, Maastricht, the Netherlands; 6 Department of Pneumology, Paracelsus Medical University, Salzburg, Austria; 7 Kaiser Permanente Center for Health Research, Portland, OR USA; 8 Department of Pulmonary Medicine, Kepler-University-Hospital, Linz, Austria; 9 Faculty of Medicine, Johannes-Kepler-University, Linz, Austria; 10 National Heart and Lung Institute, Imperial College London, London, UK; 11 Ludwig Boltzman Institute for Lung Health, Vienna, Austria

**Keywords:** Restricted spirometry, Lung function impairment, Cardiovascular disease, Hypertension, Diabetes, Comorbidity

## Abstract

**Background:**

Whether restricted spirometry, i.e. low Forced Vital Capacity (FVC), predicts chronic cardiometabolic disease is not definitely known. In this international population-based study, we assessed the relationship between restricted spirometry and cardiometabolic comorbidities.

**Methods:**

A total of 23,623 subjects (47.5% males, 19.0% current smokers, age: 55.1 ± 10.8 years) from five continents (33 sites in 29 countries) participating in the Burden of Obstructive Lung Disease (BOLD) study were included. Restricted spirometry was defined as post-bronchodilator FVC < 5th percentile of reference values. Self-reports of physician-diagnosed cardiovascular disease (CVD; heart disease or stroke), hypertension, and diabetes were obtained through questionnaires.

**Results:**

Overall 31.7% of participants had restricted spirometry. However, prevalence of restricted spirometry varied approximately ten-fold, and was lowest (8.5%) in Vancouver (Canada) and highest in Sri Lanka (81.3%). Crude odds ratios for the association with restricted spirometry were 1.60 (95% CI 1.37–1.86) for CVD, 1.53 (95% CI 1.40–1.66) for hypertension, and 1.98 (95% CI 1.71–2.29) for diabetes. After adjustment for age, sex, education, Body Mass Index (BMI) and smoking, the odds ratios were 1.54 (95% CI 1.33–1.79) for CVD, 1.50 (95% CI 1.39–1.63) for hypertension, and 1.86 (95% CI 1.59–2.17) for diabetes.

**Conclusion:**

In this population-based, international, multi-site study, restricted spirometry associates with cardiometabolic diseases. The magnitude of these associations appears unattenuated when cardiometabolic risk factors are taken into account.

## Background

Restrictive lung function is defined as reduced lung expansion expressed as a decreased total lung capacity. It may reflect several underlying conditions and diseases, such as interstitial lung diseases, pleural effusions and disorders, thoracic deformities, neuromuscular diseases, diaphragmatic disorders, obesity, heart failure, pregnancy and pain [1]. Dynamic spirometry has limitations in identifying restrictive lung diseases [2], but it can effectively exclude a restrictive disease when forced vital capacity (FVC) is normal. Further, restricted spirometry is clinically relevant as it is prevalent and associated with impaired quality of life and increased mortality [2–6].

Restricted spirometry has been associated with being female, elderly, heavy smoker, underweight or obese, and manual worker in industry [3, 7–9]. A history of tuberculosis, particularly in low- and middle-income countries where this is common is an additional risk factor [10, 11]. Several North American and European studies have reported an association of restrictive lung function with heart disease, hypertension, and diabetes [12–16]. Low FVC has also been associated with markers of cardiometabolic disease [17–20]. In addition, it has been recently shown that reduced lung function (defined as forced expiratory volume in the first second (FEV_1_) below 80% of predicted) in early adulthood is associated with a higher and earlier incidence of respiratory, cardiovascular and metabolic comorbidities later in life [21].

Within the Burden of Obstructive Lung Disease (BOLD) study, an international population-based study covering a great number of countries with different ethnic, economic and socio-cultural backgrounds, we investigated the association between the presence of restricted spirometry and the presence of self-reported physician-diagnosed cardiovascular (CVD), hypertension and diabetes, taking into account risk factors like age, sex, education, smoking and body mass index (BMI). In addition, we stratified the results for high- and low-/middle-income countries.

## Methods

### Study design and participants

The design and rationale for the BOLD initiative have been previously published [22]. A random sample population strategy was used for recruitment of participants from all study sites. In total, 33 sites (Table 1) completed data collection and were included in this analysis. Each participating site aimed to recruit a sample of at least 300 men and 300 women who were not institutionalized, were aged ≥ 40 years, and were living in a well-defined administrative area in which the total population exceeded 150,000. Participants were interviewed by means of a questionnaire and underwent a standardized assessment. Approval was obtained from each local ethics committee, and written informed consent was obtained from each participant. Participants between 40 to 89 years, with a usable postbronchodilator lung function and smoking history were extracted from the BOLD database. Those with complete data on subject characteristics, comorbidities and lung function were selected for this study.

**Table 1:** General characteristics of study participants at each site and overall

Site, Country	N	Smoking status (%)			Sex male	Age (years)		BMI (kg/m^2^)		RS (%)	Comorbidities (%)			GNI
		Current	Ex	Never	(%)	Mean ± SD		Mean ± SD			CVD	Hypertension	Diabetes	High
Adana, Turkey	806	34.9	19.9	45.3	48.3	53.6 ± 10.4		29.6 ± 5.3		14.5	11.8	27.0	10.3	No
Annaba, Algeria	862	16.7	21.9	61.4	49.8	52.9 ± 9.7		28.3 ± 5.7		26.8	6.6	22.2	14.4	No
Bergen, Norway	656	26.2	36.6	37.2	49.2	59.7 ± 12.5		26.5 ± 4.3		9.3	15.4	29.4	5.9	Yes
Blantyre, Malawi	399	3.8	9.3	87.0	39.8	52.2 ± 9.7		25.1 ± 5.4		47.6	2.5	20.1	6.0	No
CapeTown, SouthAfrica	833	46.3	21.4	32.3	37.0	54.0 ± 10.2		27.9 ± 7.4		46.5	13.3	38.9	13.2	No
Chui, Kyrgyztan	858	19.8	9.7	70.5	31.5	53.0 ± 8.8		28.5 ± 5.6		12.5	16.6	29.7	5.7	No
Colombo, Srilanka	1020	12.9	7.5	79.6	44.6	53.7 ± 9.4		24.2 ± 4.6		81.3	5.9	20.6	13.4	No
Cotonou, Benin	677	1.8	0.1	98.1	43.9	51.5 ± 9.3		26.4 ± 5.5		78.4	5.3	29.8	2.5	No
Fes, Morocco	758	8.6	18.7	72.7	46.0	55.2 ± 10.0		27.9 ± 5.3		20.2	5.8	33.1	14.6	No
Guangzhou, China	471	29.9	14.0	56.1	49.9	54.0 ± 10.6		23.3 ± 3.3		30.1	9.8	17.6	4.0	No
Hannover, Germany	681	20.7	39.4	39.9	51.2	58.0 ± 10.9		27.3 ± 4.6		9.3	17.0	38.3	6.3	Yes
Ife, Nigeria	859	2.6	7.9	89.5	39.1	55.5 ± 11.5		25.4 ± 5.4		70.7	0.2	2.3	0.8	No
Krakow, Poland	522	29.3	32.4	38.3	50.8	55.6 ± 11.4		27.7 ± 4.7		10.2	32.4	42.0	11.1	Yes
Lexington, USA	505	26.5	33.9	39.6	40.4	56.5 ± 9.8		30.8 ± 6.8		26.5	29.3	49.1	17.4	Yes
Lisbon, Portugal	709	13.3	26.8	59.9	46.7	63.3 ± 11.3		28.2 ± 4.7		10.2	17.5	37.5	11.0	Yes
London, England	672	21.0	41.2	37.8	48.1	58.0 ± 11.4		27.1 ± 5.0		16.8	7.1	33.0	6.5	Yes
Maastricht, Netherlands	589	22.9	42.4	34.6	50.8	57.5 ± 10.6		27.4 ± 4.5		10.0	17.0	29.5	7.3	Yes
Manila, Philippines	890	32.7	20.2	47.1	42.2	52.2 ± 10.1		24.9 ± 4.7		64.2	11.0	26.5	6.0	No
Mumbai, India	440	6.6	3.2	90.2	62.5	51.1 ± 8.9		23.8 ± 4.0		69.8	2.3	10.0	5.2	No
NampicuanTalugtugPhilippines	722	35.9	16.8	47.4	49.3	54.1 ± 10.5		21.5 ± 3.9		58.3	8.3	19.7	2.6	No
Naryn, Kyrgyztan	816	15.1	9.8	75.1	38.5	53.2 ± 9.7		27.0 ± 5.0		9.8	11.6	15.7	1.0	No
Penang, Malaysia	646	20.3	5.0	74.8	50.9	54.8 ± 9.3		26.0 ± 4.5		58.2	2.8	25.2	14.4	No
Pune, India	843	8.9	3.0	88.1	59.4	52.4 ± 9.8		22.1 ± 3.8		66.3	1.4	5.1	2.1	No
Reykjavik, Iceland	755	18.4	42.5	39.1	53.1	56.3 ± 11.6		27.9 ± 4.9		12.7	15.4	32.1	4.8	Yes
Riyadh, Saudi Arabia	654	7.8	17.0	75.2	54.9	50.5 ± 7.5		31.2 ± 6.0		52.1	6.7	26.6	29.4	Yes
Salzburg, Austria	1255	19.3	33.5	47.2	54.3	57.6 ± 11.3		26.4 ± 4.2		9.3	12.5	28.9	6.4	Yes
Sousse, Tunisia	658	26.7	13.2	60.0	47.0	53.0 ± 9.0		29.3 ± 5.6		26.9	5.6	21.0	10.9	No
Srinagar, India	739	10.3	1.9	87.8	54.9	51.7 ± 10.3		22.4 ± 3.6		27.9	1.4	27.1	2.2	No
Sydney, Australia	423	14.9	36.6	48.5	49.6	58.5 ± 11.9		28.0 ± 5.1		12.5	12.8	29.8	8.5	Yes
Tartu, Estonia	611	18.2	29.3	52.5	50.2	60.8 ± 12.0		28.4 ± 5.2		8.8	37.3	40.1	7.2	Yes
Tirana, Albania	926	21.8	15.2	63.0	49.8	54.7 ± 10.6		28.1 ± 4.7		17.2	4.2	22.8	6.5	No
Uppsala, Sweden	547	14.3	43.1	42.6	51.7	58.4 ± 10.9		27.0 ± 4.4		10.1	11.0	28.7	3.8	Yes
Vancouver, Canada	821	13.9	38.4	47.7	41.7	55.8 ± 11.5		26.7 ± 5.2		8.5	12.8	20.2	7.1	Yes
Total	23,623	19.0	21.1	59.8	47.5	55.1 ± 10.8		26.7 ± 5.5		31.7	10.8	26.2	8.1	

BMI, body mass index; RS, Restricted spirometry; GNI, gross national income; CVD, cardiovascular disease; SD, standard deviation

### Assessments

#### Questionnaire

Questionnaire data was obtained by face-to-face interviews conducted by trained and certified staff in the participant's native language. All participants completed a core questionnaire, based on standardised instruments, which included information on risk factors for lung disease and comorbidities [22]. A dichotomous question for self-reported physician-diagnosed comorbidities, such as heart disease, stroke, hypertension or diabetes was used. For example: “Has a doctor or other health care provider ever told you that you had *heart disease*?” In this analysis, CVD refers to the presence of either heart disease or stroke.

#### Spirometry

Lung function data were collected using the ndd EasyOne Spirometer (ndd Medical Technologies, Zurich, Switzerland) [23]. Lung function was measured before and 15 min after administration of 200 μg of salbutamol, administered with a metered dose inhaler with volume spacer. Local spirometry technicians were trained and certified. All spirograms were reviewed centrally based on standardised criteria [24]. Restrictive lung function was defined according to the lower limit of normal (< 5th percentile) of the reference values for post-bronchodilator FVC [25]. The Third National Health and Nutrition Examination Survey (NHANES III) equations were used [26].

#### Anthropometry

Body height was measured to the nearest 0.5 cm. Body weight was assessed to the nearest 0.1 kg after emptying the bladder and with the participants standing barefoot and wearing light indoor clothing. BMI was calculated as body weight/height^2^ (kg/m^2^).

### Statistical analysis

Statistics were performed in Stata, version 13.1 (Stata Corporation, College Station, TX, USA). The relation between the presence of comorbidities and restrictive lung function, and six other covariates (i.e. known cardiovascular risk factors: sex [27], education (highest level of schooling completed: less than high school, high school, some college) [28], BMI (≤ 18, 18–25, 25–30, > 30 kg/m^2^) [29], age (40–49, 50–59, 60–69, 70–89 years) [27], smoking status (never, former, current), and accumulated cigarette pack-years (0, 0–10, 10–20, 20–30, > 30)) [30] was analyzed with use of multivariable logistic regression analysis for each site. All regression models were adjusted for sampling weights within each site.

Random effects meta-analyses were performed using, for each site, the odds ratios of CVD, diabetes and hypertension, in participants with restricted spirometry compared to those without restricted spirometry. Additionally, these analyses were stratified by: (1) sex and the adjusted sex-specific odds ratios were compared for males and females by a Z-score after log-transformation; and (2) high- versus low-/middle-income countries based on gross national income per capita, according to World Bank in 2013. Study sites that reported a low number of people with a specific comorbidity (< 20) or with singularity in the data (i.e. no one with both low FVC and that specific comorbidity) were excluded from the meta-analysis because these sites could not be fitted in the model. These sites were mentioned in detail in the legends of the meta-analyses.

Differences were considered to be significant if p was less than 0.05. Heterogeneity across sites was estimated using the I^2^ statistic. I^2^ values of 0%, 25%, 50%, and 75% were respectively considered as no, low, moderate, and high heterogeneity, respectively [31].

## Results

### Study population characteristics

**Fig. 1:**
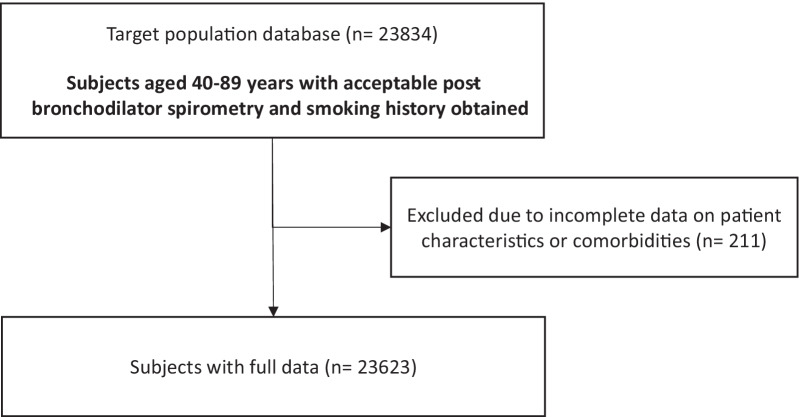
Flow chart of data extraction

From 23,834 participants aged 40–89 years, with an acceptable post-bronchodilator spirometry and smoking history, 23,623 participants had complete data with regard to subject characteristics and presence of comorbidities (Fig. 1). Fourteen out of 33 study sites (39%) were located in countries with a high-income economy. Overall and per site baseline characteristics are shown in Table 1. Study participants had a mean age of 55.1 ± 10.8 years, were slightly overweight (BMI 26.7 ± 5.5 kg/m^2^), and approximately half of them were males (47.5%). Most of them were never smokers, and one out of five were current smokers. Several sites in Africa and India had a high percentage of never smokers.

**Fig. 2:**
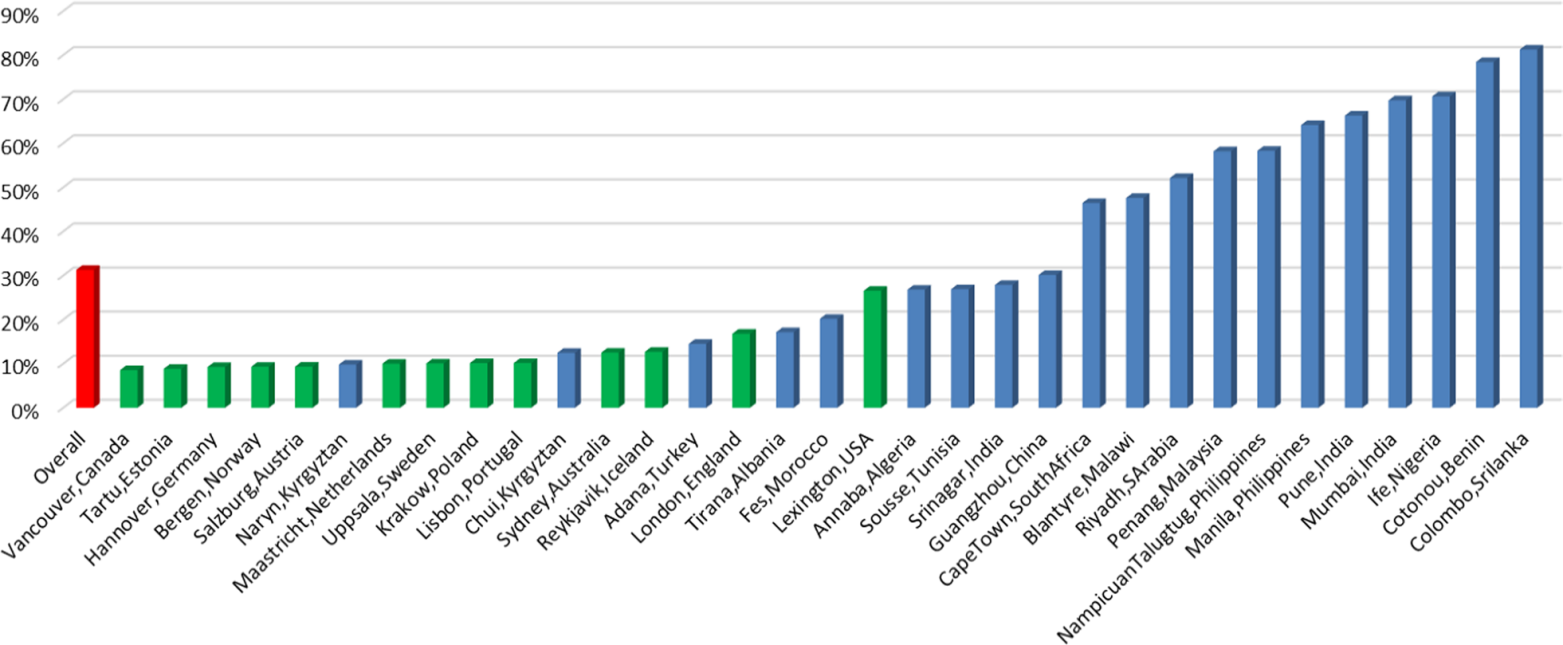
Restricted spirometry prevalence among the different sites. Prevalence of restrictive lung function for each site. Red bar gives the overall mean prevalence. Green bars indicate high-income countries, blue low-income countries

Thirty-two percent of the overall population had a restricted spirometry, with a high variation across sites. The highest prevalence of restricted spirometry was noted in Colombo (Sri Lanka) (81%), but also in sites in Africa, India, the Philippines and Malaysia had strikingly high prevalence of restricted spirometry. Of notice, 12 of 17 sites with a restriction prevalence below 20% were high income sites, compared to only 1 of the 16 sites with a restriction prevalence above 20% (Fig. 2).

### Prevalence of comorbidities

The prevalence was 10.8% for CVD, 26.2% for hypertension and 8.1% for diabetes and, was 10.9%, 28.9%, 8.2% for females, and 10.7%, 23.2%, 7.8% for males (Table 1), respectively. These comorbidities were more prevalent with older age and increasing BMI. The prevalence of CVD increased with an increasing cigarette pack-years and decreased in participants with a higher level of education. Current smokers were less likely to report CVD, hypertension or diabetes. The highest prevalence of CVD was noted in Tartu (Estonia) (37.3%), Krakow (Poland) (32.4%) and Lexington (KY, USA) (29.3%), whereas the lowest prevalence was reported in Ife (Nigeria) (0.2%), Pune (India) (1.4%) and Srinagar (India) (1.4%). The prevalence of hypertension did not differ substantially across countries with exception of Ife in Nigeria (2.3%), Pune in India (5.1%), Krakow in Poland (42.0%), Lexington, KY, in USA (49.1%) and Tartu in Estonia (40.1%). The reported prevalence of diabetes varied across sites, being lowest in Ife (Nigeria) with 0.8% and highest in Riyad (Saudi Arabia) with 29.4%.

### The association between restrictive lung function and comorbidities

Overall, participants with restricted spirometry consistently more often reported CVD (OR 1.60, 95% CI 1.37–1.86), diabetes (OR 1.98, 95% CI 1.71–2.29) and hypertension (OR 1.53, 95% CI 1.40–1.66) than those with unrestricted spirometry (Table 2). When considering sex-specific estimates, females with restricted spirometry more often reported hypertension, and diabetes, while men more often reported CVD. Regarding between-sites heterogeneity, an overall moderate heterogeneity (I^2^ 47.6%; p = 0.015) was reported for CVD and diabetes (I^2^ 47.7; p = 0.008). Non-significant low heterogeneity across sites was observed for hypertension (I^2^ 19.7%; p = 0.164).

**Table 2:** Meta-analysis of the unadjusted and adjusted odds ratios for cardiovascular disease, diabetes and hypertension in participants with restricted spirometry

Unadjusted				Adjusted		
	OR	95% CI	I^2^% and p-value for between site heterogeneity	OR	95% CI	I^2^% and p-value for between site heterogeneity
**Cardiovascular disease**						
Male	1.67	1.34–2.08	44%; p = 0.013	1.77	1.33–2.36	64.6%; p < 0.001
Female	1.56	1.26–1.93	41.7%; p = 0.015	1.52	1.20–1.93	43.2%; p = 0.011
Overall	1.60	1.37–1.86	47.6%; p = 0.003	1.54	1.33–1.79	35.2%; p = 0.038
**Hypertension**						
Male	1.49	1.30–1.69	29.4%; p = 0.062	1.56	1.37–1.78	8.6%; p = 0.329
Female	1.6	1.42–1.79	18.4%; p = 0.180	1.51	1.34–1.71	13%; p = 0.260
Overall	1.53	1.40–1.66	19.7%; p = 0.164	1.50	1.39–1.63	0%; p = 0.606
**Diabetes**						
Male	1.86	1.59–2.18	0%; p = 0.682	1.95	1.64–2.33	0%; p = 0.694
Female	1.91	1.56–2.35	44.1%; p = 0.021	1.76	1.45–2.14	26%; p = 0.145
Overall	1.98	1.71–2.29	44.7%; p = 0.008	1.86	1.59–2.17	44.9%; p = 0.008

I^2^ values of 0%, 25%, 50%, and 75% were respectively considered as no, low, moderate, and high heterogeneity. The following sites could not be included in the analysis due to a low number of participants reporting comorbidity or with singularity in the data: Blantyre (Malawi) for CVD, Cotonu (Benin) for diabetes, Guangzhou (China) for diabetes, Ife (Nigeria) for CVD, diabetes and hypertension, Mumbai (India) for CVD, Nampicuan Talugtug (Philippines) for diabetes, Naryn (Kyrgyztan) for diabetes, Penang (Malaysia) for CVD, Pune (India) for CVD and diabetes, Srinagar (India) for CVD and diabetes

**Fig. 3:**
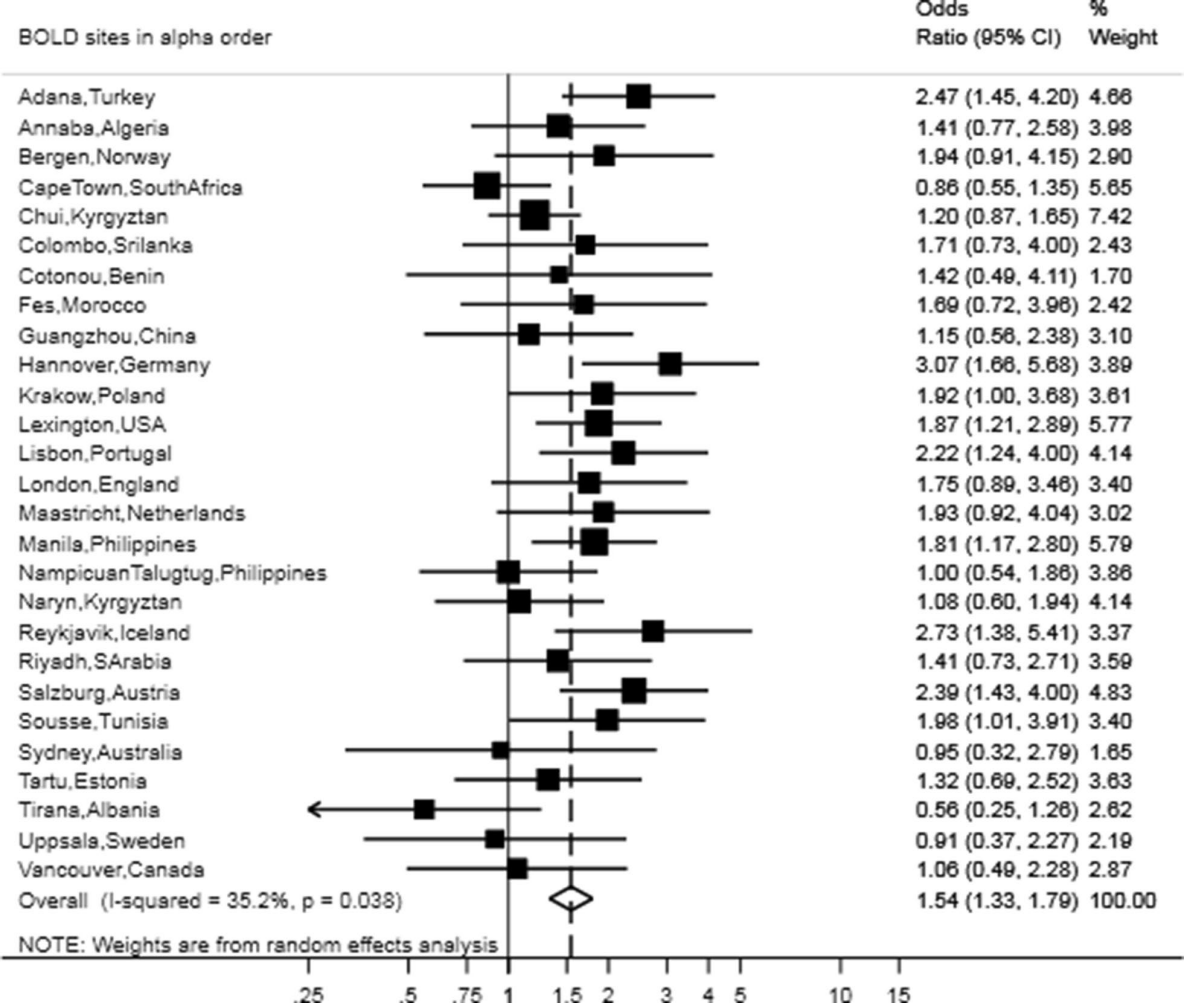
Forest plot showing the meta-analysis of odds ratios for CVD in participants with restricted spirometry compared with those without it adjusted for sex, age, BMI, smoking (pack-years and current status) and education. Heterogeneity chi-squared = 40.13, d.f. = 26 (p = 0.038). I-squared (variation in ES attributable to heterogeneity) = 35.2%. Estimate of between-study variance Tau-squared = 0.0523. Test for overall effect: Z = 5.66 (p < 0.001). The following sites could not be included in the analysis due to a low number of subjects reporting CVD or singularity in the data: Blantyre (Malawi), Ife (Nigeria), Mumbai (India), Penang (Malaysia), Pune (India), Srinagar (India)

**Fig. 4:**
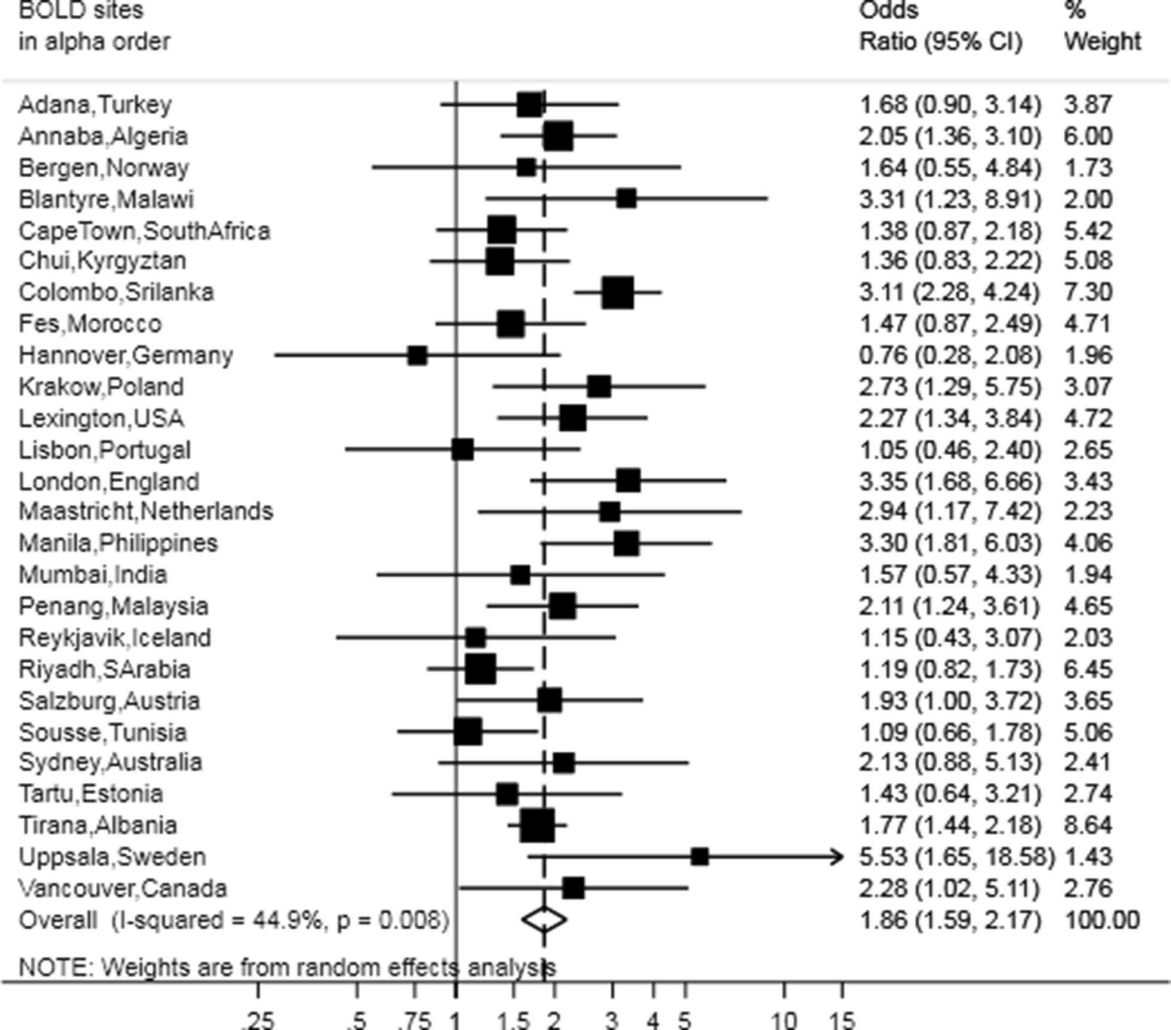
Forest plot showing the meta-analysis of the adjusted odd ratios for hypertension in participants with restricted spirometry compared to those without it adjusted for sex, age, BMI, smoking (pack-years and current status) and education. Heterogeneity chi-squared = 28.29, d.f. = 31 (p = 0.606). I-squared (variation in ES attributable to heterogeneity) = 0.0%. Estimate of between-study variance Tau-squared = 0.0000. Test for overall effect: Z = 9.94 (p < 0.001). The following sites could not be included in the analysis due to a low number of subjects reporting hypertension: Ife (Nigeria)

**Fig. 5:**
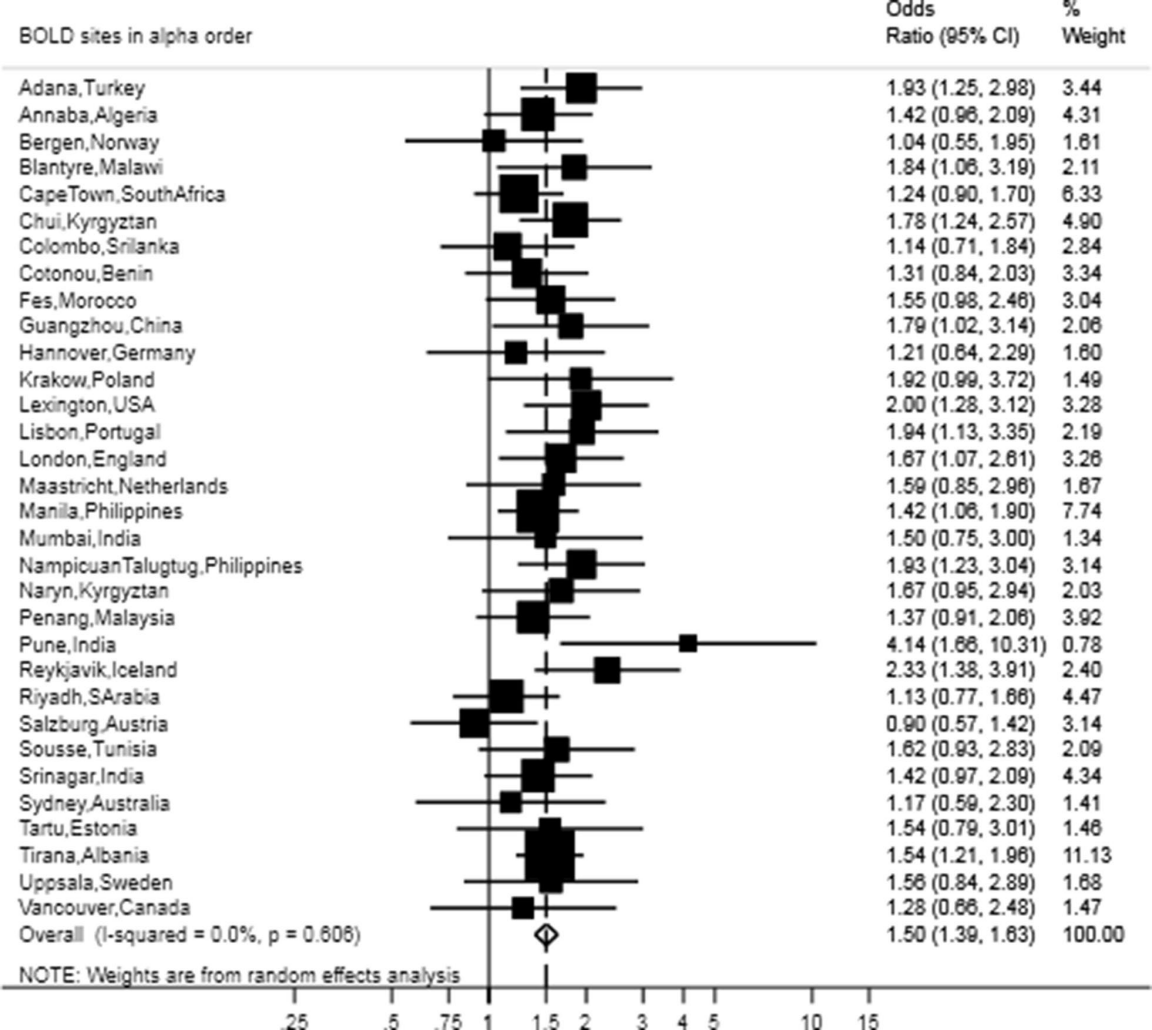
Forest plot showing the meta-analysis of the adjusted odds ratios for diabetes in participants with restricted spirometry compared to those without it adjusted for sex, age, BMI, smoking (pack-years and current status) and education. Heterogeneity chi-squared = 45.34, d.f. = 25 (p = 0.008). I-squared (variation in ES attributable to heterogeneity) = 44.9%. Estimate of between study variance Tau-squared = 0.622. Test for overall effect: Z = 7.77 (p < 0.001). The following sites could not be included in the analysis due to a low number of subjects reporting diabetes or singularity in the data: Cotonou (Benin), Guangzhou (China), Ife (Nigeria), Nampicuan Talugtug (Philippines), Naryn (Kyrgyztan), Pune (India), Srinagar (India)

After adjusting for sex, age, BMI, smoking status, pack-years and education, the presence of restrictive lung function was still strongly associated with CVD (OR 1.54, 95% CI 1.33–1.79), hypertension (OR 1.50, 95% CI 1.39–1.63) and diabetes (OR 1.86, 95% CI 1.59–2.17) (Figs. 3, 4, 5). The meta-analyses stratified by sex showed similar odds ratios as the joint analysis (Table 2).

Association of restricted spirometry with comorbidities stratified by low-/middle- and high-income countries and sex is presented in Table 3. In general, the association between the presence of restricted spirometry and comorbidities persisted in both high- and low-/middle-income countries.

**Table 3:** Meta-analysis of the unadjusted and adjusted odds ratios by sex and low- and high-income countries for cardiovascular disease, diabetes and hypertension in subjects with restricted spirometry

	Low-income countries						High-income countries					
	Unadjusted			Adjusted			Unadjusted			Adjusted		
	OR	95% CI	I^2^% and p-value for between site heterogeneity	OR	95% CI	I^2^% and p-value for between site heterogeneity	OR	95% CI	I^2^% and p-value for between site heterogeneity	OR	95% CI	I^2^% and p-value for between site heterogeneity
**Cardio vascular disease**												
Male	1.21	0.79–1.85	63.2%;	1.49	0.89–2.49	78.4%; p = 0.000	2.11	1.71–2.59	0%; p = 0.913	2.09	1.64–2.66	0%; p = 0.684
			p = 0.005									
Female	1.29	1.05–1.57	0%; p = 0.477	1.27	1.02–1.58	0%; p = 0.464	1.80	1.26–2.55	49.3%; p = 0.019	1.76	1.15–2.68	54.3%; p = 0.008
Overall	1.29	1.05–1.59	38.3%; p = 0.39	1.40	1.09–1.79	58.6%; p = 0.000	2.00	1.68–2.38	17.4%; p = 0.207	1.93	1.55–2.42	30.6%; p = 0.065
**Diabetes**												
Male	1.76	1.42–2.18	0%; p = 0.614	2.04	1.61–2.58	0%; p = 0.6014	1.99	1.57–2.54	0%; p = 0.563	1.84	1.41–2.40	0%; p = 0.562
Female	1.75	1.42–2.16	35%; p = 0.128	1.69	1.41–2.04	0%; p = 0.512	2.39	1.51–3.76	53.3%; p = 0.029	1.96	1.20–3.18	49.2%; p = 0.046
Overall	1.79	1.55–2.06	12%; p = 0.311	1.82	1.57–2.10	0%; p = 0.581	2.22	1.77–2.77	26.1%: p = 0.134	1.92	1.51–2.43	21.3%; p = 0.186
**Hypertension**												
Male	1.36	1.18–1.57	3.5%; p = 0.413	1.58	1.35–1.85	0%; p = 0.770	1.63	1.29–2.06	44.2%; p = 0.038	1.50	1.17–1.94	39%; p = 0.067
Female	1.52	1.32–1.75	26.8%; p = 0.143	1.51	1.32–1.72	4.5%; p = 0.401	1.78	1.47–2.16	0%; p = 0.462	1.50	1.15–1.95	27.1%; p = 0.164
Overall	1.45	1.31–1.61	17.1%; p = 0.187	1.53	1.39–1.70	0%; p = 0.683	1.70	1.46–1.8	26%; p = 0.105	1.50	1.25–1.80	31%; p = 0.061

I^2^ values of 0%, 25%, 50%, and 75% were respectively considered as no, low, moderate, and high heterogeneity

The following sites could not be included in the analysis due to a low number of participants reporting comorbidity or with singularity in the data: Blantyre (Malawi) for CVD, Cotonu (Benin) for diabetes, Guangzhou (China) for diabetes, Ife (Nigeria) for CVD, diabetes and hypertension, Mumbai (India) for CVD, Nampicuan Talugtug (Philippines) for diabetes, Naryn (Kyrgyztan) for diabetes, Penang (Malaysia) for CVD, Pune (India) for CVD and diabetes, Srinagar (India) for CVD and diabetes

## Discussion

In this population-based study including 33 sites from 29 countries involving more than 23,000 individuals with high-quality post-bronchodilator spirometry, restricted spirometry was associated with the prevalence of self-reported diabetes, hypertension, and CVD. These associations were barely attenuated by age, sex, smoking, BMI and education. These findings were consistent across sites, regardless of gross national income.

We found a high variation across sites in prevalence of restrictive spirometry, with higher prevalence in low-/middle-income countries (particularly Asian and African countries). We purposively used the NHANES III prediction equations for Caucasians for all sites [26, 32]. The use of locally derived reference equations from our study sample would prevent to reveal all environmental influences. Literature supports that differences in ethnicity have minor influence on lung development in relation to environment [5]. Use of race- or ethnicity-based predictive equations is controversial, and assumptions that observed variations in lung function are due to race or ethnicity should be avoided [33]. Rather, studies such as BOLD highlight the marked differences in developmental and environmental exposures across populations that affect lung growth and mature lung function. In the BOLD study, for example 15.4% of participants in Cape Town (South Africa) and 10.8% in Manila (Philippines) (reported prevalence of FVC below the lower limit of normal (LLN) 46.5% and 62.4%, respectively) self-reported a history of tuberculosis [10, 34], and the actual prevalence of tuberculosis in those regions may be higher.

In line with our results, several other population-based studies consistently reported the positive association between restrictive lung function and cardiovascular diseases [14, 35]. Lindberg et al. measured cardiovascular disease in subjects with restricted, obstructed and normal lung function. They reported a lower prevalence of CVD in restricted than in obstructed participants, but a higher prevalence in restricted than in healthy persons [14]. Eriksson et al. reported that heart disease among subjects with restrictive lung function was about three to six times more prevalent compared to those with normal spirometry. The proportion of hypertension in restrictive subjects was similar to that in subjects with Chronic obstructive pulmonary disease (COPD) stage 3 and 4 [35]. Previous research also demonstrated that the risk for experiencing a cardiovascular event in subjects with restricted lung function during 15-years follow-up was similar to subjects with moderate airflow limitation indicating COPD [12].

In the above studies, a restrictive spirometry pattern has been commonly described as a decreased FVC in combination with a normal or increased FEV1/FVC ratio, and then compared to an obstructive spirometry pattern independent of FVC. This likely ignores the potential coexistence of a restriction in vital capacity in subjects with obstructive lung function. More severe static hyperinflation related to severe airflow limitation might result in a decreased vital capacity, but this is uncommon in a population-based study. We previously showed that the association of CVD and hypertension with airflow limitation in this study population was largely explained by age and smoking habits and that the adjusted risk for diabetes was even lower in subjects with airflow limitation [36]. Here we aimed to focus on the association between low FVC and cardiometabolic comorbidities independent of the presence or absence of airflow limitation and showed a much stronger association for restrictive spirometry with cardiometabolic comorbidities compared to our previous findings related to obstructive spirometry. Hence, previous reported associations of cardiometabolic comorbidities with obstructive spirometry in population studies might be partly explained by coexistent restriction.

It has been suggested that poor nutritional status is a risk factor for impaired lung function [37, 38]. In a longitudinal population study, Ubilla et al. reported that low BMI in adults associates with low FEV_1_ and FVC. In the present study, of the 10 countries with the lowest BMI, 8 had a restricted spirometry prevalence of over 40%, which all were low-/middle- income countries. On the other hand, obesity has been associated with decreased vital capacity in several observational studies [14, 39, 40]. In this study, we observed lower FVC in countries with a high prevalence of obesity, like the United States of America. Also, significant association between restrictive lung function and diabetes has been shown [3, 41]. A potential explanation for the association between low FVC and the presence of diabetes could be through obesity and related insulin resistance. Nevertheless, even after adjustment for BMI, the adjusted odds ratio did not alter the significant association in the present study. In line with this, the presence of diabetes has been shown to be associated with a restrictive lung function pattern in a meta-analysis [42]. Furthermore, subclinical impairment of lung function was seen in children with type 1 diabetes mellitus and was associated with disease duration and the degree of metabolic control [43]. This might not surprise as the large vascular network and high collagen and elastin composition of the pulmonary system, is prone to microvascular damage and nonenzymatic glycation in diabetes.

There is increasing attention for the potential role of early life events and their role in lung function and other organ function development. Restricted lung function could be the result of suboptimal lung development, related to environmental influences in utero, during early childhood and adolescence. Prenatal foetus’s exposure, thereafter childhood environmental exposure, respiratory infections and nutritional influences are potential contributing factors to suboptimal lung development. Agusti et al*.* described the abnormal lung development and suggested that lower lung function, might be related to suboptimal development of the other organs, which might relate to an earlier and higher prevalence of comorbidity [21, 44]. This is a potential explanation for the association seen in the current study between low lung function and the presence of comorbidities.

The stratified results for low-/middle- and high-income countries yielded comparable outcomes for hypertension and diabetes, although the associations between comorbidities and restricted spirometry were less strong in low-/middle-income countries. This could be related to the high proportion of subjects with restrictive lung function in low-/middle-income countries which could dilute the effect size. On the other hand, the effect of a lower socioeconomic status on underestimating the prevalence of non-communicable diseases has been reported to be more pronounced in countries with low-income economies compared to those with high-income economies [45].

In high income countries, in which the NHANES III reference equations might be more applicable, the main objective and outcome of this study, that restrictive lung function is strongly associated with cardiometabolic comorbidities is confirmed.

By its cross-sectional design, the current general population study describes the association between restricted spirometry and cardiometabolic comorbidity. The strictly standardized spirometry is one of the main strengths of the current study. Another strength of this study is that a great number of population-based samples worldwide, accounting for regional differences with regard to exposure [46] and potential ethnical and socioeconomical differences were investigated. Remarkably, the meta-analyses had low to moderate heterogeneity across study sites. Limitations of the current study include healthy participant bias and the self-reported nature of the comorbidity assessment. As discussed, we purposively used the same prediction equations for all countries as NHANES III [26]. The use of locally derived reference equations from our study sample would prevent to reveal all environmental influences.

## Conclusion

In conclusion, the results of the international BOLD study show that on the population level, subjects with restricted spirometry are more often affected by CVD, hypertension and diabetes. Furthermore, this association is not attenuated by the presence of common risk factors, such as aging and smoking. These findings emphasize the urgent need to understand better the mechanisms underlying the association between impaired lung function and cardiometabolic disease, particularly in low-income countries where restrictive lung function is more prevalent and a shift from communicable diseases to non-communicable diseases is underway.

## Data Availability

The datasets used and analyzed during the current study are available from the corresponding author on reasonable request.

## Abbreviations

BMI: Body Mass Index
BOLD: Burden of Obstructive Lung Disease
CI: Confidence Interval
COPD: Chronic Obstructive Pulmonary Disease
CVD: Cardiovascular disease
FEV_1_: Forced expiratory volume in the first second
FVC: Forced vital capacity
LLN: Lower limit of normal
NHANES III: The Third National Health and Nutrition Examination Survey
OR: Odd ratio
